# Genetically Predict Diet‐derived Antioxidants and Risk of Neurodegenerative Diseases Among Individuals of European Descent: A Mendelian Randomization Study

**DOI:** 10.1002/brb3.70766

**Published:** 2025-08-12

**Authors:** Qing‐Qing Duan, Wei‐Ming Su, Xiao‐Jing Gu, Jiang Long, Zheng Jiang, Kang‐Fu Yin, Wei‐Chen Cai, Bei Cao, Li‐Yi Chi, Xia Gao, Ju‐Rong Li, Yong‐Ping Chen

**Affiliations:** ^1^ Department of Neurology, Centre for Rare diseases, West China Hospital Sichuan University Chengdu Sichuan China; ^2^ Mental Health Center, West China Hospital Sichuan University Chengdu Sichuan China; ^3^ Department of Neurology First Affiliated Hospital of Air Force Military Medical University Xi'an Shanxi China; ^4^ Department of Geriatrics Dazhou Central Hospital Dazhou Sichuan China

**Keywords:** antioxidant, Mendelian randomization, neurodegenerative diseases, oxidative stress

## Abstract

**Background:**

The incidence of neurodegenerative disorders (NDDs) is increasing, and currently, there are no curative treatments available for these conditions. The potential benefits of supplementation with diet‐derived antioxidants and their metabolites for NDDs remain a subject of debate. In this study, we employed a two‐sample Mendelian randomization (MR) analysis to investigate the potential causal relationship between elevated circulating antioxidant levels and the risk of NDDs.

**Methods:**

We used single‐nucleotide polymorphisms (SNPs) related to diet‐derived antioxidants and *p* < 1E‐05 as instrumental variables (IVs). The NDDs we studied included Parkinson's disease (PD) (33,674 cases and 449,056 controls), Alzeimers disease (AD) (111,326 cases and 677,663 controls), amyotrophic lateral sclerosis (ALS) (27,205 cases and 110,881 controls), and frontotemporal dementia (FTD) (3526 cases and 9402 controls) from GWASs conducted in the European descent. Two‐sample MR was performed together with a series of sensitivity analyses. The main statistical analyses were conducted using the package “TwoSampleMR (V.0.5.6)” in R (V.4.2.0).

**Results:**

Genetically predicted levels of α‐tocopherol and carotene were found to be associated with a reduced risk of ALS, with odds ratios (OR) of 0.45 (95% confidence interval [CI]: 0.31, 0.66; *p* = 3.97 × 10^‐5) and 0.82 (95% CI: 0.68, 0.99; *p* = 0.0427), respectively. Similarly, vitamin E (OR 0.70; [95% CI 0.50, 0.98]; *p * = 0.0358) and ascorbate (OR 0.85; [95% CI: 0.73, 0.98]; *p* = 0.0216) demonstrated a protective effect against PD. Furthermore, elevated levels of retinol were associated with a reduced incidence of FTD, with an OR of 0.92 (95% CI: 0.86, 0.99; *p* = 0.0196), although they were also linked to an increased risk of ALS (OR 1.02 [95% CI 1.01, 1.04], *p* = 0.0017) and PD (OR 1.06 [95% CI 1.02, 1.09], *p* = 0.0121). No significant causal association was observed between circulating antioxidants and AD.

**Conclusion:**

This MR study indicated a potential protective effect of α‐tocopherol and carotene on ALS, vitamin E and ascorbate on PD, and retinol on FTD, while also identifying retinol as a risk factor for ALS and PD. These findings could contribute to the exploration of dietary therapies for NDDs. Further research is necessary to substantiate the potential associations between diet‐derived antioxidants or their metabolites and the risk of NDDs in individuals.

## Graphical abstract

Genetically predicted levels of α‐tocopherol and carotene are inversely associated with the risk of amytropic lateral sclerosis (ALS); vitamin E and ascorbate levels demonstrate an inverse relationship with the risk of Parkinson's disease (PD). Additionally, retinol levels may decrease the risk of frontotemporal dementia (FTD), albeit with an associated increased risk of ALS and PD.

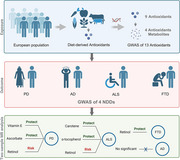

## 1 Introduction

Neurodegenerative diseases (NDDs) are a major health problem for the world's aging population. NDDs are an extensively heterogeneous group of diseases that affect the nervous system and are one of the most common causes of disability worldwide (Heemels 2016). The prevalence of NDDs is rapidly increasing, leading to a substantial rise in associated social and economic burdens (Hou et al. 2019, GBD 2019 Dementia Forecasting Collaborators 2022, Nandi et al. 2022). Common NDDs include Parkinson's disease (PD), Alzheimer's disease (AD), amyotrophic lateral sclerosis (ALS), and frontotemporal dementia (FTD). Currently, the diseases can only be treated symptomatically but cannot be reversed (Stephenson et al. 2018). Risk factors for NDDs include aging, and genetic and environmental factors (Stephenson et al. 2018, Cannon and Greenamyre 2011, Niccoli and Partridge 2012, Lin and Beal 2006), but the definite cause is currently unknown. Therefore, it is important to identify the risk factors of NDDs and reveal the underlying pathogenesis, which could inform the development of primary prevention.

Several common mechanisms, including oxidative stress, are involved in the pathogenesis of NDDs (Ransohoff 2016, Golpich et al. 2017, Singh et al. 2019). A balanced state of oxidative stress is essential for proper body function. Inefficient oxidative phosphorylation may generate reactive oxygen species (ROS), affecting normal function, especially for organs sensitive to ROS, such as the brain (Yun et al. 2020). In many cases of NDDs (such as AD, PD, ALS, and FTD), elevated levels of ROS and reactive nitrogen species (RNS) and alterations in the antioxidant defense system are common (Teleanu et al. 2022, Perluigi et al. 2024, Korczowska‐Łącka et al. 2023, Beers et al. 2022, Dong‐Chen et al. 2023, Trist et al. 2019, Keating et al. 2023). In the NDDs model, many antioxidants treatments displayed beneficial consequences, such as CoQ10, vitamin E, vitamin C, selenium, and KC14 peptide (Ibrahim Fouad 2020, Komaki et al. 2019, De Nuccio et al. 2021, Zeng et al. 2020, Umapathy et al. 2024, Vijayanand et al. 2024). Antioxidants might diminish oxidative stress‐induced damage by scavenging free radicals and are the potential reagents for preventing and treating NDDs (Esmaeili et al. 2022, Elfawy and Das 2019).

The cellular antioxidant system that prevents tissue damage is composed of endogenous and exogenous antioxidants (Pisoschi and Pop 2015). Previous studies have shown that diet‐derived exogenous antioxidants can inhibit the generation of ROS and repair other oxidized scavengers (Ballaz and Rebec 2019, Lee and Ulatowski 2019, Duester 2008). Some studies have reported the benefits of diet‐derived exogenous antioxidant supplementation on NDD risks (Lee et al. 2020, Silvestro et al. 2021, Schepici et al. 2020, Pohl and Kong Thoo Lin 2018, Yoritaka et al. 2021, Cruz‐Aguilar et al. 2021), whereas some researche has reported a null effect (Park et al. 2011, Fischer et al. 2018, Noguchi‐Shinohara et al. 2014). This inconsistency may arise because observational studies and randomized controlled trials (RCT) are susceptible to confounding factors and reverse causality. Additionally, RCTs are not only costly but also require long‐term follow‐up and large‐scale cohort studies to provide more reliable evidence (Msaouel et al. 2023).

Mendelian randomization (MR) is an alternative method to infer causality of lifelong risk factors (exposure) on diseases (outcome) using genetic variants as instrumental variables (IVs). MR design eliminates the influence of confounding factors because alleles are randomly distributed during gamete formation and conception (Davey Smith and Ebrahim 2003). Therefore, the results of MR avoid reverse causality and confounding bias (Verduijn et al. 2010).

Hence, in this study, we used MR analysis to assess the correlation between diet‐derived antioxidants or their metabolites and the risk of NDDs, yielding a list of diet‐derived antioxidants that can be intervened with and utilized to guide the prevention and management of NDDs.

## 2 Method

### 2.1 Study Design

**FIGURE 1:**
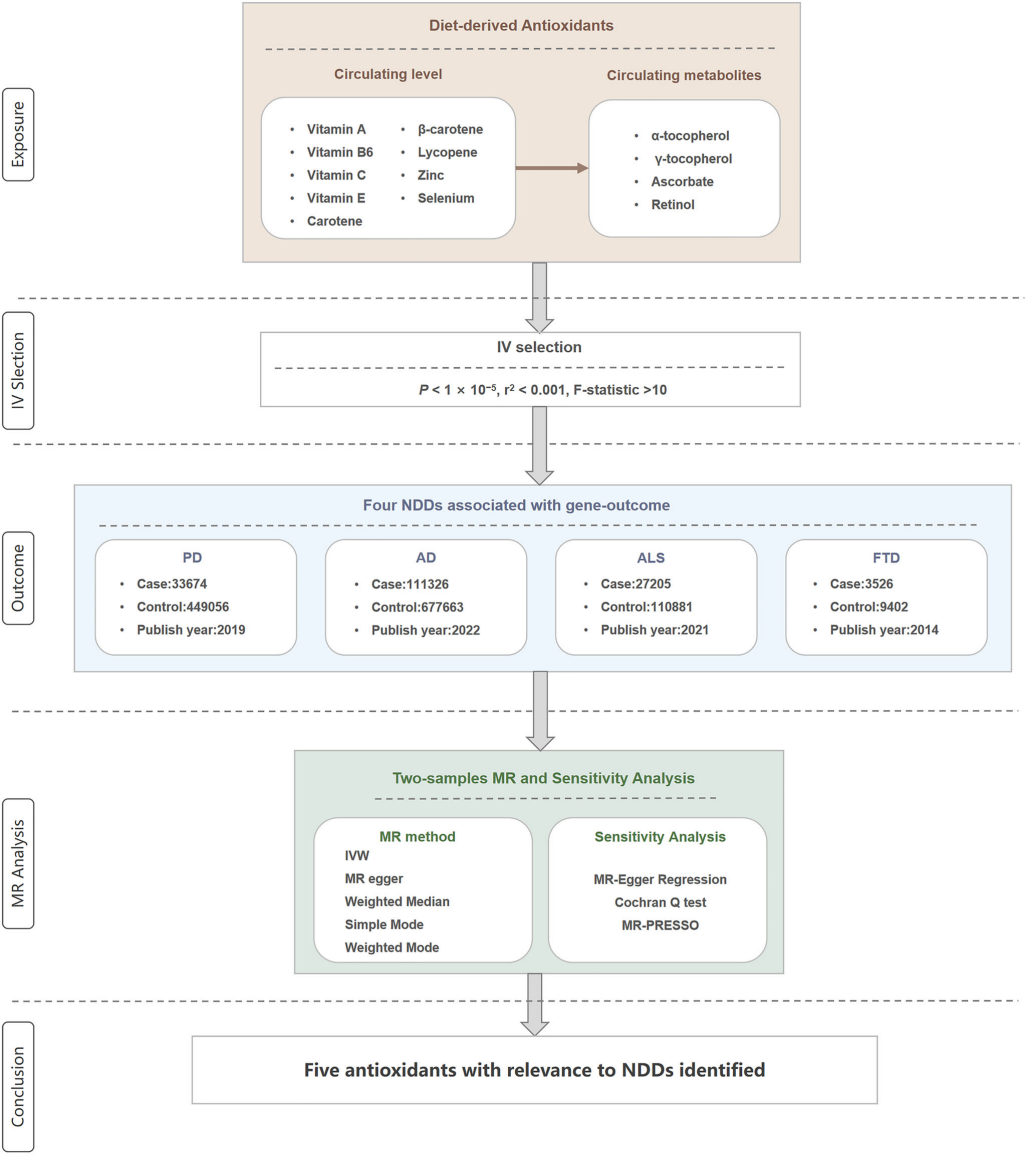
**The flow chart of this study**. It summarizes the antioxidants studied, the identification of genetic instruments, the selection of outcome data, and the MR and sensitivity analysis methods. **Abbreviation**: MR, Mendelian randomization.

A two‐sample MR was performed to explore the association between diet‐derived antioxidants and NDDs. We first identified IVs of 13 diet‐derived antioxidants as exposures by searching genome‐wide association studies (GWAS) from relevant articles or publicly available websites. Then, four GWAS of NDDs were selected as outcomes. Both exposure and outcome cohorts were restricted to individuals of European ancestry to reduce bias from population stratification. Finally, a range of sensitivity analyses were conducted to verify the reliability and stability of the results. The flowchart of the study is given in Figure 1.

### 2.2 Selection of Genetic Instrumental Variables

Overall, this study focused on major dietary sources of antioxidants. It examined both the absolute levels of antioxidants measured directly in blood and the relative concentrations of their corresponding circulating metabolites quantified in plasma or serum. Specifically, for absolute antioxidant levels, we identified vitamin A, vitamin B6, vitamin C, vitamin E, carotene, β‐carotene, lycopene, zinc, and selenium (Luo et al. 2021, Gunderson et al. 2021, Bjørklund et al. 2022). For antioxidant metabolites, we used α‐tocopherol, γ‐tocopherol, ascorbic acid, and retinol.

We performed a search on the IEU openGWAS (https://gwas.mrcieu.ac.uk/) and GWAS Catalog (https://www.ebi.ac.uk/gwas/) for published GWAS related to the antioxidants mentioned above. When multiple GWASs existed for a single trait, we selected the GWAS with the largest sample as the exposure. We systematically curated genome‐wide significant SNPs associated with 13 diet‐derived antioxidants from different GWAS (Supplement Table 1). To ensure the authenticity and accuracy of the conclusions on the causal link between the diet‐derived antioxidants and NDDs risk, the following quality control steps were used to select the optimal IVs. First, independent SNPs (*r^2^* < 0.001) related to exposures were gained. Second, set an appropriate threshold to select the IVs. The first threshold selected SNPs less than the genome‐wide statistical significance threshold (*p* < 5 × 10^−8^) to serve as IVs. Unfortunately, after we selected SNPs, only a small number of diet‐derived antioxidants were selected as IVs, and to explore more relations between diet‐derived antioxidants and NDDs to obtain more comprehensive results, we used the second threshold that identified SNPs that were smaller than the locus‐wide significance level (*p* < 1 × 10^−5^) and selected them as the IVs set to find more potential causal associations, except plasma β‐carotene and circulating lycopene. (The completed raw GWAS of these two indexes were not provided, so we used the LD and *p*‐value as reported in the original study [β‐carotene (Hendrickson et al. 2012): LD < 0.2, *p* < 5 × 10^−8^; lycopene (D'Adamo et al. 2016): LD < 0.001; *p* < 5 × 10^−6^]). Third, to ensure a strong correlation and minimize bias from weak IVs, we considered an *F*‐statistic of at least 10 as sufficient for performing an MR analysis, which is well‐accepted in the field (Palmer et al. 2012, Jiang et al. 2023). Summarized characteristics of diet‐derived antioxidants were shown in Supplementary Table S1.

### 2.3 GWAS Summary Statistics of NDDs

Given that AD and PD are the most common NDDs (Scheltens et al. 2021, GBD 2016 Neurology Collaborators 2019), and considering the disability and fatal nature of ALS and FTD, we conducted research on these four NDDs. As mentioned above, to avoid demographic heterogeneity, only aggregated European population data were included. When multiple GWASs existed for a single trait, we selected the GWAS with the largest sample as the outcome (Supplementary Table S1). The PD database (33,674 cases and 449,056 controls) was obtained from the International Parkinson's Disease Genomics Consortium (IPDGC), including three previously reported GWAS studies, 13 new datasets, and UKB proxy‐case data (excluding 23andMe) (Nalls et al. 2019). The summary statistics of Alzheimer's Disease were extracted from the European Alzheimer & Dementia Biobank (EADB) consortium, which consists of 111,326 clinically diagnosed and “proxy” AD cases and 677,663 controls from 15 European countries (Bellenguez et al. 2022). Summary statistics of ALS were obtained from the most recent and largest ALS GWAS, which contained 10 million SNPs in 138,086 European individuals (27,205 ALS cases and 110,881 controls) (Van Rheenen et al. 2021). Regarding the selection of FTD data, we selected FTD GWAS data with a sample size of 12928 published in 2014 (3,526 cases and 9402 controls) (Ferrari et al. 2014). The subtype of FTD will be re‐analysed if the sample size is more than 500, so behavioral variant FTD (1377 cases and 2754 controls) was also included (Ferrari et al. 2014).

All studies had been approved by a relevant ethical review board, and participants had given informed consent. Ethical approval was not required because of the public characteristics of the data of GWAS.

### 2.4 MR Design and Sensitivity Analysis

MR analyses were first conducted using the inverse variance weighted (IVW) method (Burgess et al. 2013). This method consisted of meta‐analyzing SNP‐specific Wald ratios between the effect outcome and exposure using a random‐effects inverse variance approach, where each ratio is weighted according to its standard error while accounting for potential heterogeneity in the measurements (Zheng et al. 2017). In addition, we also used MR Egger, weighted median, simple mode, and weighted mode to assess the causal relationship between 13 antioxidants and four NDDs. The IVW method is reported to be slightly more powerful than the others under certain conditions (Bowden et al. 2016). Therefore, the results were mainly based on the IVW method, with the other four methods serving as complements. Results are expressed as odds ratios (ORs) and 95% confidence intervals (CIs) on NDDs risk. All analyses were performed using R version 4.2.1 statistical software (R Foundation for Statistical Computing, Vienna, Austria). In addition, MR analyses were performed using the R‐based package “TwoSampleMR (V.0.5.6).”

We conducted a sensitivity analysis to assess the following points: (1) whether the assessment results are robust and the conclusions are reliable; (2) whether the results are potentially biased (e.g., horizontal pleiotropic, heterogeneity); (3) whether there is a particular instrumental variable that significantly affects the outcome variable. We applied MR‐PRESSO (Verbanck et al. 2018) and MR‐Egger regression (Bowden et al. 2015) tests to monitor the potential horizontal pleiotropy effect. For each single SNP, the MR‐PRESSO outlier test calculates the *p*‐value for pleiotropic effects, while the MR‐PRESSO global test assesses the overall significance of pleiotropy. SNPs are ranked based on their *p*‐values from the outlier test in ascending order and removed sequentially. After each SNP is removed, the MR‐PRESSO global test is reapplied to the remaining SNPs. This process continues recursively until the global test *p*‐value becomes non‐significant (*p* > 0.05). The final list of SNPs, after excluding those with significant pleiotropy, is used for subsequent MR analysis. Furthermore, heterogeneity was quantified using Cochran's *Q* statistic, and *p* < 0.05 was considered to indicate heterogeneity. In addition, leave‐one‐out analysis to assess whether MR might be influenced by a single SNP with a particularly large horizontal pleiotropic effect. To visualize the results, we plotted the leave‐one‐out analysis, the funnel plot, and the scatter plot. To account for multiple testing corrections, the Bonferroni method was applied. Bonferroni correction controls the Type I error probability in multiple tests by adjusting the significance level, and the significance level for each test is α/n, where n represents the number of tests and α is the originally set significance level. By this means, the overall Type I error rate of the entire set of hypothesis tests can be ensured not to exceed α (Curtin and Schulz 1998). According to the formula mentioned above, the significance threshold for four NDDs and one FTD subtype was set to 7.7E‐4 (0.05/65, 13 exposures × 5 outcomes = 65 tests).

## 3 Result

### 3.1 Causal Effects of Diet‐derived Antioxidants on NDDs

We analyzed the causal correlations between 13 antioxidants and 4 NDDs. Among 13 exposures examined in this MR investigation, α‐tocopherol was significantly associated with ALS (OR 0.45 [95% CI 0.31, 0.66], *p* = 3.97E‐05). The analysis results using all five MR methods are shown in Supplementary Tables 2–7.

**FIGURE 2:**
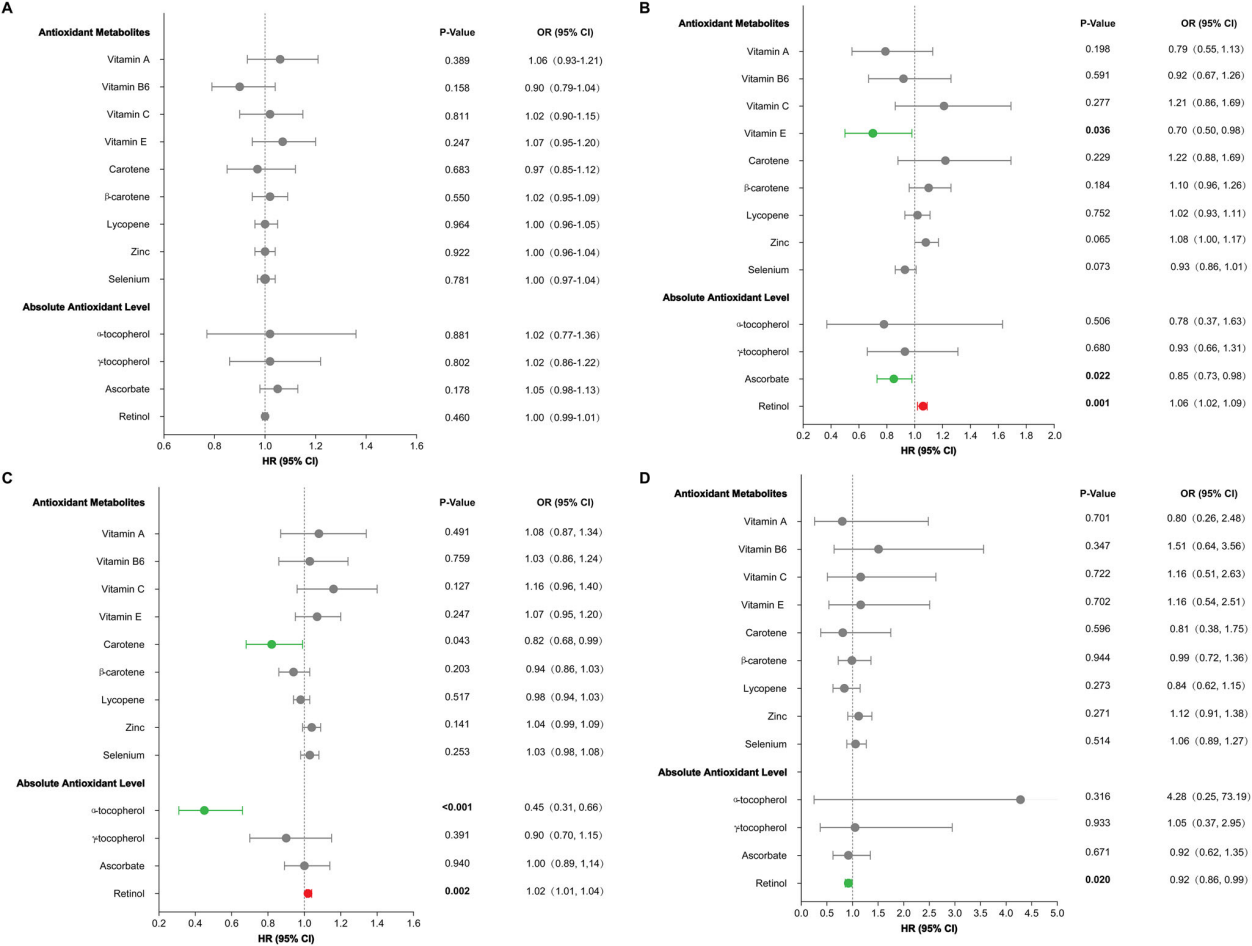
**ORs for the genetically predicted association between circulating antioxidants and NDDs were plotted as forest plots. (A)** MR result of antioxidants and AD, **(B)** MR result of antioxidants and PD, **(C)** MR result of antioxidants and ALS, and (**D)** MR result of antioxidants and FTD. Forest plot showing the protective antioxidants (green), risk antioxidants (red), and nonsignificant antioxidants (grey). All estimates were calculated based on the random effects IVW method, where dots represent the OR and horizontal bars represent the 95% confidence intervals.

We also identified the potential correlation between vitamin E and PD and carotene and ALS, respectively. Genetically predicted vitamin E was associated with lower odds of PD (OR 0.70 [95% CI 0.50, 0.98], *p* = 0.0358), and carotene showed evidence of the protective effect on ALS (OR 0.82 [95% CI 0.68, 0.99], *p* = 0.0427). For antioxidant metabolites, ascorbate is associated with a reduced incidence of PD (OR 0.85 [95% CI 0.73, 0.98], *p* = 0.0216). Retinol was also associated with a lower incidence of FTD (OR 0.92 [95% CI 0.86, 0.99], *p* = 0.0196). However, a positive association between retinol and ALS (OR 1.02 [95% CI 1.01, 1.04], *p* = 0.0017) and PD (OR 1.06 [95% CI 1.02, 1.09], *p* = 0.0121) was identified (Table 1 and Figure 2). However, these correlations are not significant after the Bonferroni correction.

**TABLE 1:** Genetically correlation between long‐term, genetically increased circulating antioxidants and NDDs risk.

Exposure	Outcome	No.SNP	MR‐Analysis		Cochran *Q* test		MR‐Egger		MR‐PRESSO
			OR (95%CI)	*p*	*Q* value	*p*	Intercept	*p*	*p*
Vitamin E	PD	33	0.70 (0.50, 0.98)	3.58E‐02	42.13	0.11	0.00927	0.58	0.14
Carotene	ALS	25	0.82 (0.68, 0.99)	4.27E‐02	26.62	0.32	−0.00608	0.50	0.31
α‐tocopherol	ALS	11	**0.45 (0.31, 0.66)**	**3.97E‐05**	6.23	0.80	0.00194	0.88	0.81
Ascorbate	PD	14	0.85 (0.73, 0.98)	2.16E‐02	12.66	0.47	0.00781	0.55	0.57
Retinol	ALS	77	1.02 (1.01, 1.04)	1.67E‐03	59.80	0.91	0.00189	0.68	0.93
	PD	89	1.06 (1.02, 1.09)	1.21E‐03	125.42	5.43E‐03	−0.00593	0.56	4.00E‐03
	FTD*	46	0.92 (0.86, 0.99)	1.96E‐02	23.03	1.00	−0.02002	0.52	0.99

**Abbreviations**: ALS, amyotrophic lateral sclerosis; FTD* frontotemporal dementia of all subtypes; OR, odds ratio**;** PD, Parkinson's disease; SNP, single nucleotide polymorphism.

### 3.2 Sensitivity Analysis

We used Cochran's *Q* test, MR‐Egger interpretation, and MR‐PRESSO approaches to conduct sensitivity analyses on antioxidants. *Q*‐test results suggested significant heterogeneity between retinol and PD (*p* = 0.005). Except for the association between retinol and PD (MR‐PRESSO *p* = 0.004), there was no evidence of horizontal pleiotropy existing for other antioxidants according to the MR‐Egger intercept and MR‐PRESSO global test. In addition, we identified rs1842947 as an outlier SNP in the MR‐PRESSO outlier test for retinol and PD (*p* < 0.089), and then we found that the outlier correction results had a similar range and direction to the raw analyses (distortion test *p* = 0.588). In the leave‐one‐out analyses, we found that the risk for four NDDs with each antioxidant exposure remained essentially stable and consistent after excluding one SNP at a time. To visualize the results, we plotted the leave‐one‐out analysis and the scatter plot, and all graphics are shown in **Supplementary Figures** 1 and 2.

## 4 Discussion

The study assessed the relationship between diet‐derived antioxidants and four NDDs using MR. Based on large‐scale GWAS, our study provides new, objective evidence for the relationship between higher α‐tocopherol and a lower risk of ALS. In addition, several antioxidants had suggestive effects against NDDs, including vitamin E, carotene, ascorbate, and retinol. However, our study did not support the association of vitamin A, vitamin B6, vitamin C, β‐carotene, lycopene, zinc, selenium, and γ‐tocopherol with candidate NDDs that we are concerned about.

The present MR study found strong evidence of the association of α‐tocopherol with decreased risk of ALS, which is consistent with the results of prior research (Michal Freedman et al. 2013). α‐tocopherol, as a peroxide‐free radical scavenging antioxidant, can inhibit lipid peroxidation mediated by free radicals (Jiang et al. 2022). Moreover, studies have shown that α‐tocopherol can reduce lipid peroxidation caused by lipopolysaccharide (LPS), microglia, and interleukin‐6 (IL‐6) (Shibata et al. 2006, Godbout 2004), and it also has a positive impact on neuroplasticity (Hernangomez et al. 2014). A study evaluated the changes induced in the transcriptional profile of NSC‐34 motor neurons treated with α‐tocopherol and found that α‐tocopherol may be an efficacious therapy in preventing motor neuron death (Chiricosta et al. 2019). A previous study also suggested that tocopherol derivatives can reduce muscle atrophy and improve survival in SOD1^G86R^ mouse models by protecting against oxidative and DNA damage‐induced stresses (Von Grabowiecki et al. 2015). A clinical trial indicated that patients receiving riluzole plus α‐tocopherol were less likely to progress from the milder to the more severe condition of the ALS Health State scale (Desnuelle et al. 2001). These findings, together with our MR result, suggest that α‐tocopherol may be an independent protective factor for ALS. For the first time, we identified α‐tocopherol as an independent protective contributor to ALS independently of the influence of vitamin E.

In this study, carotene was associated with a lower risk of ALS. Carotene is a fat‐soluble substance that provides neuroprotection by inhibiting neuroinflammation, microglial activation, regulation of autophagy, reduction of oxidative damage, and activation of defensive antioxidant enzymes (Manochkumar et al. 2021). Mitochondrial dysfunction is involved in the complex pathophysiology of ALS (Cunha‐Oliveira et al. 2024), and carotene regulates mitochondrial ROS, inhibits mitochondrial fission, promotes mitochondrial fusion and biogenesis, and improves mitochondrial function (Ademowo et al. 2024). A previous prospective cohort study indicated that carotenoids are protective against ALS, which is consistent with what we found (Fitzgerald et al. 2013). Meanwhile, a diet rich in carotenoids has been shown to have a protective effect on the function of ALS patients (scored by ALSFRS‐R) (Nieves et al. 2016). Carotene is classified as α‐, β‐, and γ‐carotene and lycopene (Ávila‐Román et al. 2021), and our MR investigation suggested that total major carotene intake has a significant association with ALS, whereas β‐carotene and lycopene alone do not. The GWAS for β‐carotene and lycopene had small sample sizes, which may affect the reliability of MR results, and therefore validation in a larger sample GWAS is needed.

The analysis results show that vitamin E and ascorbate were identified to be associated with PD. In contrast to the exogenous lipophilic vitamins present in the body, vitamin E exists universally in cell membranes and is in the highest concentrations throughout the body. Therefore, vitamin E is thought to play an important role in regulating redox interactions in vivo (Miyazawa et al. 2019). Furthermore, it has been recently confirmed that in the PINK1 knockout model of Parkinson's disease, vitamin E has the ability to restore the plasticity of the cerebral striatum (Schirinzi et al. 2019). In line with our results, previous case‐control studies suggested that plasma vitamin E levels were lower in PD patients compared to controls (Chen et al. 2009). Ascorbate, the metabolite of vitamin C, is the most important antioxidant in the central nervous system, playing a role in neuron differentiation and maturation, regulation of neuron apoptosis, involvement in myelin formation, and regulation of cholinergic, catecholaminergic, and glutamatergic systems (Ferrada et al. 2020, Travica et al. 2017). Furthermore, ascorbate plays a role in the epigenome mechanism of gene expression (Young et al. 2015), and abnormal concentrations of epigenetic markers 5‐methylcytosine and 5‐hydroxymethylcytosine in the brain are associated with the development of neurodegenerative diseases, including PD (Kwon et al. 2016, Kaut et al. 2019). Our study suggests that high concentrations of ascorbate may reduce the risk of PD, perhaps because of its ability to reduce the production of ROS (Travica et al. 2017, Giordano et al. 2014). However, it is difficult to draw conclusions from this result, as the sample size of the ascorbate GWAS was small, and a larger GWAS for ascorbate is needed to better clarify its association with PD. Earlier research has shown that vitamin C deficiencies are common in PD patients (Medeiros et al. 2016) and that vitamin C supplementation can reduce oxidative damage in animal models (Man Anh et al. 2019, Khan et al. 2012). Thus, their results differed from our MR study, where genetically elevated ascorbate should decrease the risk of PD while vitamin C does not. The correlation between vitamin C and PD was not significant as expected, suggesting that different substances from the vitamin C family may play different pathological roles in PD, which needs further exploration in larger sample sizes and sophisticated clinical trials. In contrast, our data suggested a potential causal association between elevated levels of retinol and a higher risk of PD. However, sensitivity analysis results indicate significant heterogeneity, and causal conclusions are less reliable, suggesting the presence of significant outliers (which may represent pleiotropic variation) or when evidence for causal effects depends on one or a few variants.

Vitamin A is also called retinol or all‐trans‐retinol, which is an antioxidant. One active metabolite of vitamin A, retinoic acid (RA), is involved in neuroplasticity and immune regulation (Janesick et al. 2015). Vitamin A, through its metabolites acting as ligands for ligand‐dependent transcription factors, regulates the expression of multiple genes involved in oxidative stress responses, thereby exerting indirect transcriptional control and enhancing the body's antioxidant defense mechanisms (Blaner et al. 2021). In the current MR study, genetically predicted concentrations of circulating retinol were associated with a lower risk of FTD, and these findings were robust to sensitivity MR methods. Although there is a lack of research examining a clear relationship between vitamin A and FTD, vitamin A and cognitive function have been studied. The prefrontal cortex (PFC) and its connections to the mid‐dorsal thalamus are critical for cognitive flexibility and working memory, and RA signaling plays a key role in the development and evolutionary expansion of the PFC (Shibata et al. 2021). In addition, the controlled synthesis of RA is critical for regulating synaptic plasticity in brain regions involved in learning and memory, such as the hippocampus. Higher levels of retinol and carotenoids are associated with better cognitive performance in humans (Wołoszynowska‐Fraser et al. 2020). Our analysis also provides new evidence for the role of retinol in the risk of ALS development, which was consistent with the finding that dietary administration of retinoic acid worsened symptoms and shortened lifespan in the SOD1^G93A^ mouse model (Crochemore et al. 2009), but inconsistent with the result that using high‐affinity retinoid agonists showed a protective potential in the same mouse model (Riancho et al. 2016). Therefore, the dose and the metabolism of retinol might be a modifying factor in ALS, and our findings still need to be corroborated in prospective studies.

In our study, we found that there was no significant causal association between the levels of vitamin A, vitamin B6, vitamin C, β‐carotene, lycopene, zinc, selenium, and γ‐tocopherol that we included and the risk of candidate NDDs. Our findings were obtained in the population of food intake antioxidants alone or low‐dose supplementation, suggesting that the route and dose of administration need to receive more attention or be a more critical intervention when further studies of antioxidants are undertaken.

The main strength of this study is the use of MR methods for exploring the genetic association between diet‐derived antioxidants and the risk of NDDs, which overcomes the short duration of antioxidant exposure in RCTs and reduces potential confounding bias in observational studies. Using a two‐sample design and a large‐scale GWAS allows for analysis with sufficient power to detect small effects of altered exposure. In addition, we used the GWASs for antioxidants with little overlap (<10.0%) with outcome GWASs to guarantee the lowest type 1 error rate.

Nevertheless, our study had several limitations. Firstly, the GWAS data of NDDs come from the European population, lacking data support from other regions. Therefore, it is still necessary to further explore whether our conclusion is applicable to the global scale. Secondly, the GWAS data of NDDs we used failed to conduct stratified analysis based on the covariates of interest (such as age, gender, smoking, drinking, and underlying diseases) or the lack of specific antioxidants in the population. Thus, it is impossible to determine whether supplementing antioxidants in certain subgroups can affect the risk of NDDs. Third, the two‐sample MR model has several general limitations, including the assumption of linear association, lifetime effect estimates, and the potential for inference bias due to genetic phenomena, such as variation or confounding by canalization and LD between sample substructures, which may lead to false positives or false negatives. Recent research has suggested that future studies should adopt an integrated approach by combining multiple omics platforms to deeply understand the pathogenesis of diseases within the context of the complex interplay between genes and environment over time. (Agustí et al. 2022) Finally, the GWAS samples included in our study are relatively small in size, which may lead to a decline in the detection ability of gene‐phenotype associations. Therefore, it is necessary to conduct GWAS on a larger population scale and real‐world studies based on large samples to enhance the reliability and universality of the results.

## 5 Conclusion

In conclusion, this MR study elucidates that genetically predicted levels of α‐tocopherol and carotene are inversely associated with the risk of ALS, while vitamin E and ascorbate levels demonstrate an inverse relationship with the risk of PD. Additionally, elevated circulating retinol levels may decrease the risk of FTD, albeit with an associated increased risk of ALS and PD. It is imperative to further investigate the underlying mechanisms driving these associations, as such insights are likely to inform novel strategies for the prevention and treatment of NDDs.

## Author Contributions

**Qing‐Qing Duan**: methodology, software, visualization, and writing – original draft. **Wei‐Ming Su**: software. **Xiao‐Jing Gu**: conceptualization. **Jiang Long**: validation. **Zheng Jiang**: validation. **Kang‐Fu Yin**: writing – review and editing. **Wei‐Chen Cai**: validation. **Bei Cao**: funding acquisition. **Li‐Yi Chi**: validation, writing – review and editing. **Xia Gao**: writing – review and editing, and validation. **Ju‐Rong Li**: validation, writing – review and editing. **Yong‐Ping Chen**: conceptualization, funding acquisition, writing – review and editing, validation, and project administration.

## Consent

The authors have nothing to report.

## Conflicts of Interest

The authors declare no conflicts of interests.

## Peer Review

The peer review history for this article is available at https://publons.com/publon/10.1002/brb3.70766

## Supporting information




**Supplementary Tables**: brb370766‐sup‐0001‐Tables.xlsx

**Supplementary Figures**: brb370766‐sup‐0001‐Figures.docx

## Data Availability

The datasets analyzed in this study are publicly available summary statistics. Data used can be obtained upon a reasonable request to the corresponding author.
