# Supplementary material for: Genetically Predict Diet‐derived Antioxidants and Risk of Neurodegenerative Diseases Among Individuals of European Descent: A Mendelian Randomization Study
Source: Brain Behav. 2025 Aug 12;15(8):e70766. doi: 10.1002/brb3.70766 (PMC12340713; doi:10.1002/brb3.70766)
Supplement: Supplementary file 2 — Supplementary Figures: brb370766‐sup‐0001‐Figures.docx [file BRB3-15-e70766-s002.docx]

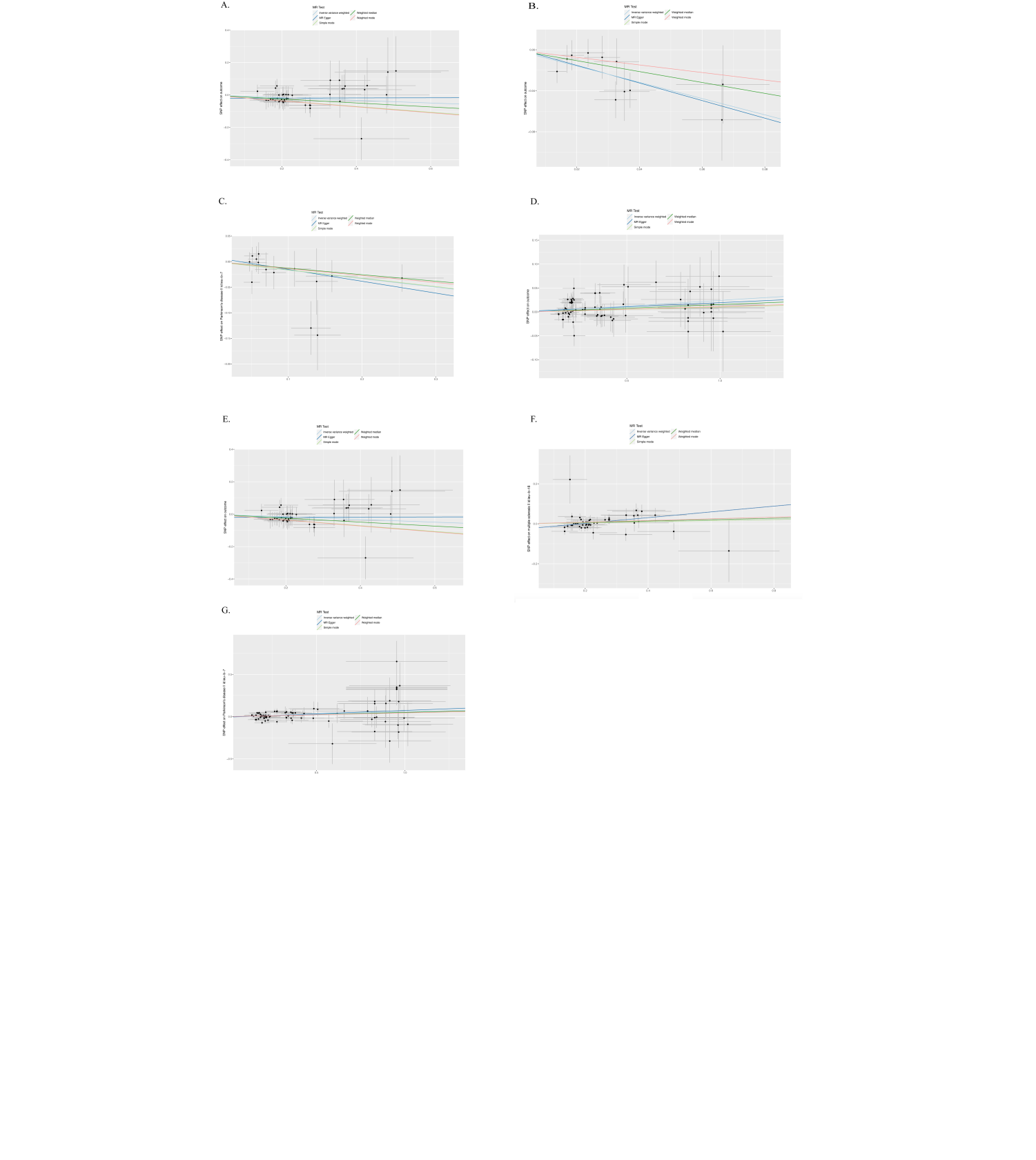


**Supplement Figure 1. Scatterplot of meaningful causality.** A: scatter plot for the association of circulating vitamin E with PD. B: scatter plot for the association of absolute circulating α-tocopherol with ALS. C. scatter plot for the association of circulating ascorbate with PD. D: scatter plot for the association of circulating retinol with ALS. E: scatter plot for the association of circulating retinol with PD. F: scatter plot for the association of circulating carotene with ALS. G: scatter plot for the association of circulating retinol with FTD-META

.


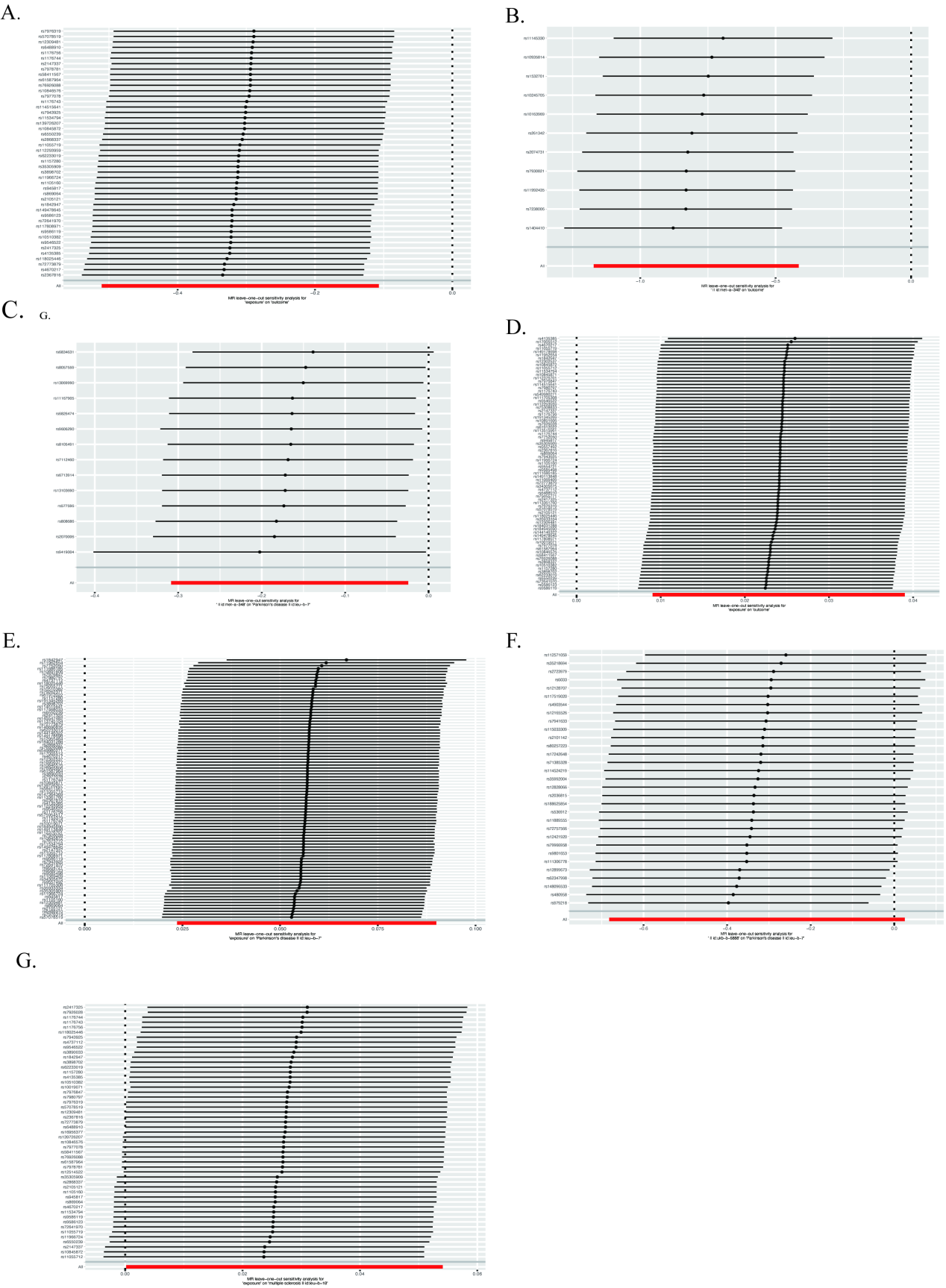


**Supplement Figure 2. leave-one-out permutations of meaningful causality.** A: leave-one-out permutations for the association of absolute circulating vitamin E with PD. B: leave-one-out permutations for the association of circulating α-tocopherol with ALS.C: leave-one-out permutations for the association of circulating ascorbate with PD. D: leave-one-out permutations for the association of circulating retinol with ALS. E: leave-one-out permutations for the association of circulating retinol with PD. F: leave-one-out permutations scatter plot for the association of circulating carotene with ALS. G: leave-one-out permutations for the association of circulating retinol with FTD.
